# Cumulative Effects, their Causal Pathways and Social Impact Assessment: Lessons from Aotearoa New Zealand

**DOI:** 10.1007/s00267-025-02232-z

**Published:** 2025-07-23

**Authors:** C. Nicholas Taylor, Michael Mackay

**Affiliations:** 1 Nick Taylor and Associates, Rangiora, New Zealand; 2 Lincoln University, Faculty of Environment, Society and Design, Lincoln, New Zealand

**Keywords:** Cumulative effects assessment, social impacts, causal pathways, irrigation, New Zealand

## Abstract

Cumulative effects result incrementally from a series of actions over time. On their own, the causative actions behind cumulative effects may be one-off and minor but become significant when they aggregate. Drawing on a practice example from Aotearoa New Zealand—the widespread expansion of irrigation over thirty years and associated land use changes—this paper extends our understanding of cumulative effects in several ways. First, the example shows that in Aotearoa New Zealand, the institutional and practice aspects of cumulative effects assessment are poorly developed, despite being required in legislation and recognized in case law. Part of the problem, we argue, is an unclear mandate for strategic environmental assessment that has limited the development of consistent cumulative effects assessment theory and practice. The “practice” element to improve, we suggest, is an integrated assessment of accumulating impact pathways, with particular implications for the conduct of social impact assessments. The key “institutional” improvement needed is a stronger role for strategic environmental assessment that incorporates social impact assessment, to address the social impacts of cumulative effects in advance, as well as for monitoring actual effects.

## Introduction

There is growing global interest in cumulative effects, how they interact and the ways they can be assessed and managed, as reflected in the emerging conceptual and practice-based literatures (e.g., Blakely and Franks 2021 and IAIA 2017, respectively). In this paper we consider how social impact assessment (SIA) is required for full assessments of cumulative effects and their social outcomes.

Cumulative effects result incrementally, in space and over time from a series of actions (including polices and plans), projects, or activities. On their own, the causative actions behind cumulative effects may be one-off and minor but become significant when they aggregate. These types of impacts might not be anticipated fully when considered on their own as the impacts of a single proposal. They can, however, be anticipated when an impact assessment process is designed to do so (Canter and Ross 2010).

In many contexts, including Aotearoa New Zealand (NZ), the institutional and practice aspects of cumulative effects assessment (CEA), while required in legislation, are poorly developed, despite extensive experience with impact assessment generally. Furthermore, while the role of strategic environmental assessment (SEA) in addressing cumulative effects is articulated in the literature, the lack of a clear and consistent framework for SEA, in NZ at least, may have slowed the development of CEA (Morgan and Taylor 2021).

Using the example of irrigation development and associated land use changes in one NZ region, we show that project and regional level cumulative effects can result in multiple impact chains that create unanticipated social outcomes. We argue these outcomes require strategic thinking and assessment by authorities tasked with planning, regulation, and environmental permits or consents. In NZ impact assessment, practice is often focused on single environmental vectors with insufficient integration between practice types (Mackay et al. 2024).

Furthermore, as in many other jurisdictions, the example we discuss reinforces that there is a need to look further into cumulative effects, to their impact chains, and their effects on people and communities, in terms of human health, and social wellbeing (Grace and Pope 2021; Malakar et al. 2023). It also shows, as per Arnold et al. (2024), that Indigenous groups have a particular concern about the need to consider social impacts as part of CEA. This assessment process is complex because of the intervening processes that require our attention, including climate change, biodiversity loss, social inequalities, and the undermining of human rights. An integrated assessment process is required to deal with this complexity.

We consider two potential sources for cumulative effects. First are those arising from proximate projects in the same receiving environment. These can be similar projects with similar effects or different projects with similar effects (e.g., cumulative effects evident from proximate wind farms). We note also that successive or incremental expansion from a single project can also result in cumulative effects. Second are more diffuse sources of cumulative effects from aggregated changes, such as urban incursion into productive farmland, multiple hospitality and tourism developments in a destination area, and the outcomes of new intensive farming in a catchment, the example we focus on in this paper.

These diffuse examples are usually analyzed, considered and responded to strategically at a sectoral, sub-regional or regional level as is the case with the example of regional water management that we describe. Our example shows that impact assessment practitioners need to consider these diffuse effects strategically at the local (catchment) level and at the regional level while integrating SIA into their assessments, with SEA an essential tool for addressing cumulative effects in an integrated way through the development and testing of regional policies and plans.

## Cumulative Effects and Strategic Responses to them

We reviewed existing knowledge about CEA from two points of view. The first looks at the links between CEA and SEA, and the integration of SIA into these approaches. The second considers issues around CEA and SEA in NZ, a country with a long history of impact assessment practice, including SIA (Taylor and Mackay 2016), but less explicit application of SEA (Morgan and Taylor 2021).

### Linking CEA and SEA, and Implications for the Integration of SIA

Cumulative effects are well recognized in impact assessment practice, and their assessment is often promoted to improve the focus of SEA on sustainability outcomes (Bragagnolo et al. 2012). Jones (2015), in a substantial review, notes that cumulative effects are aggregated, collective, and accruing changes to ecosystems that are open and almost endlessly complex. However, while CEA is commonly focused on valued environmental components, cumulative effects can have much broader societal implications, through disruptions to social systems or human health (Canter and Ross 2010) and consequences for human wellbeing (Malakar et al. 2023). This complexity makes CEA a rather challenging task, as acknowledged in the literature (Morgan 2012; Grace and Pope 2021).

SEA is an integral part of impact assessment theory and practice, seen as an important tool alongside project-level assessment (Morgan 2012) that adds a broader temporal and spatial perspective on the environment, including cumulative effects. SEA injects a greater degree of anticipatory thinking into policy and plan making processes that encourage and support integrated approaches to spatial planning and resource management, usually within an over-arching sustainability and adaptive management framework (Canter and Ross 2010; Ehrlich 2022). Greater attention to potential cumulative effects in SEAs informs decisions about, for example, the separation of activities in space or time, bio-physical and social carrying capacities, levels of acceptable change, and the setting of environmental standards.

SEA has a particular role in considering cumulative effects at a regional scale. Noble (2008), for example, advanced the importance of using a strategic assessment framework to guide the assessment and management of cumulative effects for an ecological region in Canada. He notes the interest at that time in examining and managing cumulative effects at a regional scale. Peeters et al. (2022) also consider potential cumulative effects, and their causal pathways, at a bioregional spatial scale in Australia, while Jenkins (2018; 2019) argues for a strategic response for improved water management in NZ. A strong strategic assessment framework for areas such as catchments, districts or regions ensures there is ongoing monitoring, alongside policy criteria and agreed thresholds (set environmental limits) that guide the full assessment and mitigation of effects in project and strategic assessments.

To achieve this strategic framework, it is necessary to conceptualize how cumulative effects of one or more bio-physical factors can aggregate, with consequences for people and communities. We found there is increasing evidence of CEA practice considering the linkages between cumulative effects in bio-physical systems and the consequent changes in social and cultural systems. Based on case studies of indigenous CEAs, Arnold et al. (2023, 2024), for example, note the poor integration of SIA and cultural assessments with CEA. They provide guidance on better practice in Canadian jurisdictions around an integrated approach in project and regional settings, for enhanced sustainability outcomes.

As with other forms of impact assessment, SIA is focused commonly on disciplinary-driven variables, for example population change, employment opportunities, changes in community organization, and public health and safety (Burdge 2004), rather than the aggregated effects from multiple sources, including bio-physical effects. By considering where cumulative effects result in multiple chains of effects with social impacts, it is possible to understand the consequences for social wellbeing such as for human health, sense of place and amenity values. This approach requires SIA practitioners to understand how sets of impacts, successively or in combination, can combine to cause cumulative effects on social wellbeing through webs and chains of effects (Loxton et al. 2013; Ehrlich 2022; Peeters et al. 2022; Taylor and Mackay 2022).

There is a longstanding call for better consideration of the interplay of bio-physical and social-economic causes and effects (Slootweg et al. 2001). More recently, Ehrlich (2022), argues that to address complex issues of sustainability, impact assessment requires integrated analysis across expert areas. The complexity of cumulative effects requires a collaborative learning process that drives community conversations about outcomes within an SEA framework (Mackay et al. 2024).

The international literature on CEA practice identifies several issues. Foremost is this need for better integration of bio-physical and social-cultural analysis from the scoping stage of assessment using integrative frameworks (Arnold et al. 2023, Mackay et al. 2024). There is also a need to identify better the physical and temporal scales of an assessment, with an emphasis on work at larger scales such as catchments and regions, as discussed in the example in this paper. Also, scoping is important for establishing temporal frames for cumulative effects including the relevance of legacy issues (environmental, social and cultural). Then, there are clear calls for monitoring and IA follow-up across projects and change sources (Arnold et al. 2023).

### Cumulative Effects Assessment in NZ

We now turn to the experience with CEA in NZ, an early adopter of environmental procedures in 1973, with wide use of impact assessment since then. This South Pacific country (est. population 5.1 m in 2022) is highly urbanized yet remains dependent economically on natural resource sectors of primary production and tourism, while promoting a “clean green” image. A sophisticated system of environmental management and impact assessment should lead to sustainable and effective resource management with strong positive outcomes for human wellbeing.

The Ministry for the Environment and Statistics NZ (2022) considered 50 updated, environmental indicators and found increasing pressures across key environmental problems such as intensification of land uses, loss of productive soils, pollution of waterways, damage or loss of indigenous biodiversity, and inadequate consideration of adaptations to climate change. There is demand for greenfield land for housing and vigorous debates about urban form, especially in the face of climate change. These pressures are despite impact assessment being well established in an integrated system of environmental decision making from local to national levels (Morgan and Taylor 2021). However, practice has not lived up to the potential of the system, especially in the absence of strong guidance on how to address cumulative effects and confusion about cumulative effects in case law (Olagunju et al. 2021). There is evidence of a deteriorating environment and increasing community concern about cumulative effects, even of aggregated green energy sources such as wind farms (Baines and Taylor 2021).

Cumulative effects are listed but not well defined in New Zealand’s environmental legislation, the Resource Management Act (RMA), 1991(s2) which says effects are:

- any positive or adverse effect; and
- any temporary or permanent effect; and
- any past, present, or future effect; and
- *any cumulative effect which arises over time or in combination with other effects regardless of the scale, intensity, duration, or frequency of the effect*, and also includes
- any potential effect of high probability; and
- any potential effect of low probability which has a high potential impact.

Under the Act, district and regional councils prepare policies and plans for managing environmental outcomes. The Purpose of the Act (s5) requires them to do so to “promote the sustainable management of natural and physical resources” that “enables people and communities to provide for their social, economic and cultural wellbeing”.

Legal interpretations of the Act are critical in establishing these local principles for the practice of IA, including CEA. Appeals on council decisions on policies, plans and resource consents are heard by the Environment Court (the Court) and then to higher courts on points of law. The Court commonly judges evidence on impacts but Olagunju et al. (2021, p.27) comment that there has been “an almost total separation of the legal discourse from the impact assessment literature” on cumulative effects assessment.

An early decision in the New Zealand Court of Appeal had a strong influence on the interpretation of cumulative effects. That decision viewed cumulative effects as those that will result from the activity for which the resource consent was being sought, either through gradual build up over time (e.g., water pollution), or as combinations of effects. Crucially, the Court viewed the potential effects of other possible activities that might be proposed in the same area in the future as being outside the definition of cumulative effects. This does not accord with how the IA literature defines cumulative effects, and subsequent legal commentary in NZ. Some decisions have moved away from that narrow view, towards one that recognizes the effects of multiple activities over space and time (Olagunju et al. 2021). Because of these legal confusions, approaches to the practice of CEA have varied with limited mention of cumulative effects in many environmental assessments.

In a review of this practice, Milne (2008, p.14) referred to the possible collective significance of multiple small actions as “death by a thousand cuts” that have temporal and spatial dimensions. These cumulative effects require sufficient environmental assessment and monitoring in an adaptive approach. In this approach, decisions made under the RMA, such as on land uses and project consents, are taken under policies and rules established in land use and natural resource plans, guided by National Policy Statements and/or National Environmental Standards produced by central government, as outlined by Morgan (2022). International practice of impact assessment clearly recognizes that effects can arise and accumulate in a locality either from a series of similar actions over time, or from a series of different actions with the same effect in a locality. In theory, therefore, the assessment of cumulative effects in NZ should occur during policy and plan preparation as well as in project (resource consent) assessments.

Because of these evident institutional, legal and practice issues, there has been little practice experience with cumulative effects assessment, and few NZ guidelines to draw on. There is, however, an increasing understanding of cumulative effects, at least at the project level. Baines and Taylor (2021) identified the social impacts from proximate windfarms, and increasing resistance from residents faced with an additional proposed project. Another project example was a proposed open aggregates mine (quarry) close to Christchurch City, where quarrying was needed to supply aggregates for rebuilding after a sequence of destructive earthquakes in 2010–11. A proposed quarry was sited close to several proximate quarries, another under construction, and others consented yet undeveloped. Under the existing case law, consented but unbuilt quarries form part of the environmental baseline (Taylor 2017).

Technical evidence presented to the Court (Borthwick 2017) identified potential cumulative effects of the quarry across several vectors including dust, noise, heavy traffic on local roads and visual amenity. The social impact evidence (Taylor 2017), along with evidence from residents, showed these physical effects of an additional quarry had potential social impacts on the people living in the surrounding rural-residential area. The Judge noted in her decision to refuse consent that the primary concern of the Court was the cumulative effects of the proposed quarry on the rural character of the area, and its consequent amenity values to residents. She acknowledged that on their own effects such as noise, dust, heavy transport on local roads, and landscape change, may well be minor with appropriate plans for mitigation and management in place, but they had the potential to be cumulative given the number of quarries already permitted in the area (Borthwick 2017).

**Fig. 1:**
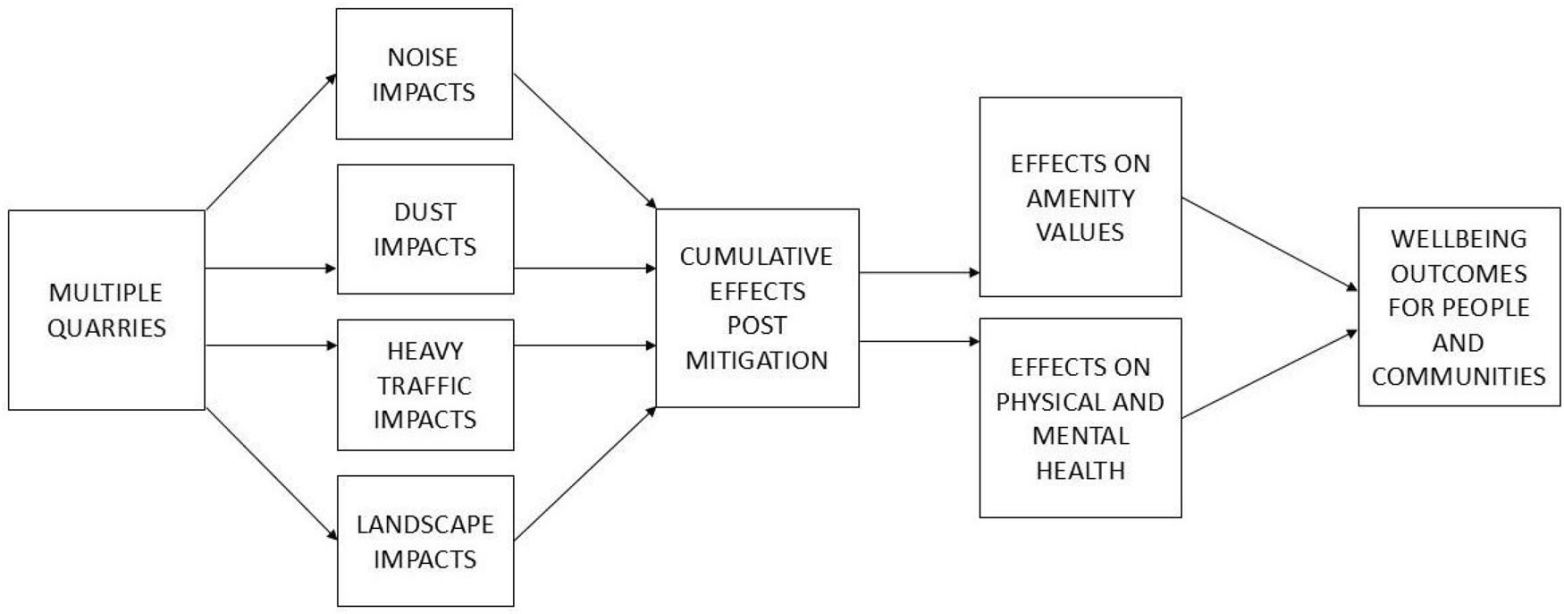
Local cumulative effects of proximate quarries on people and communities

An important insight from this case is that cumulative environmental effects on several vectors could aggregate to have consequences for the wellbeing of people and communities in linked impact chains (Fig. 1) based on a series of unpublished diagrams used to develop social evidence for the Court. This method of webbing and chaining, driving all forms of impact to their social consequences, is described in Taylor et al. (2004). When a series of cumulative effects from proximate quarries aggregate and interact, they potentially result in deterioration of the local residential living environment from the loss of amenity values and from effects on the respiratory and mental health of residents.

Agencies in NZ making policy and plans, or processing approvals for several activities generating similar effects in proximity to each other, need to recognize better that these combined actions can result in cumulative effects, and consider how to respond through policies and plans that establish standards and rules around land uses for the siting of projects.

Turning now to cumulative effects from distributed activities at a regional scale, our review found strong arguments for better directed assessments of cumulative effects with stronger institutional foundations. Two government-funded National Science Challenge programs in NZ have addressed the institutional and practice issues around cumulative effects, in the coastal-marine environment (Davies et al. 2018) and in the freshwater environment (Mackay et al. 2024). These interdisciplinary research projects identified the need for better conceptual frameworks for understanding how cumulative effects are understood and assessed in these environments. They call for better understanding of changes from baseline conditions, clearer definition of scale and significance when determining effects, and collaborative sharing of knowledge systems including indigenous Māori knowledge. In practice, the policy response often seems to be too little and too late, and improved, collaborative, strategic assessment is required to tackle substantial problems such as settling limits around the use and quality of freshwater (Taylor and Mackay 2016), as explored in the following example.

## An Example of Intensive Agriculture and Waterways

A regional scale example is provided here, examining the cumulative effects of intensive agriculture under irrigation in the alluvial plains of the Canterbury region (Canterbury Plains), South Island, NZ. The impacts in this example arise from the widespread expansion of irrigation, new farming technologies, and associated changes in land use from predominant sheep production, mixed cropping, and cropping farming to predominant dairy production. In this example we explore the background to irrigation and land-use change in the region, regulatory reforms taking place, and the SIAs of sub-catchments as part of those reforms. From these SIAs, we identify consequential social impacts from cumulative environmental effects and their multiple impact pathways, and we discuss the methods used to assess them.

### Irrigation and Agricultural Intensification

The Canterbury Region (area 45,000 square kilometers/17,000 square miles) features the Plains, which lie between alpine areas (the Southern Alps) and the sea (Pacific Ocean). These plains are intersected by several major rivers and have a significant groundwater resource underlying their alluvial gravels.

The Canterbury economy relies heavily on a mix of pastoral farming, cropping, and horticulture that uses these water resources for irrigation and discharges nutrients and contaminants into them. Agricultural intensification—which has been evident on the Plains since the 1960s (MacLeod and Moller 2006)—accelerated considerably from the 1990s with the conversion of many dryland farms to more intensive, irrigated uses, such as dairy farming. This land-use change included a technological shift from flood irrigation to spray systems such as center pivots, allowing more efficient use of water and increased production. The considerable capital costs of headworks, and modern irrigation systems, on farms, precipitated land-use intensification that raised farm incomes, employment on and off farm, and business activity (Pangborn & Woodford, 2011).

Official agricultural statistics for the Canterbury Region illustrate the magnitude of land-use change over the past two decades. Between 2002 and 2022, the expansion of irrigation infrastructure enabled the region’s dairy farming area to nearly double, increasing from 241,000 hectares to 480,000 hectares (StatsNZ 2024a). Over the same period, the number of dairy cows in Canterbury grew tenfold, rising from 113,000 to 1.3 million (StatsNZ 2024b). The changes in question occurred across all major Canterbury catchments, from the Waitaki River in the south to the Hurunui River in the north.

### Cumulative Effects of Land-use Change

In less than 30 years, the cumulative effects from multiple, aggregated on and off farm actions of land use change with irrigation, resulted in direct and indirect impacts on surface and groundwater, including lower and more variable flows in rivers and streams from water abstractions, changes in stream-bed morphology, changes in stream ecology, and reduced water quality from increased levels of nutrients and bacterial contamination. The result was significant public concern, a variety of political responses and a period of regulatory reforms, which utilized a strategic and collaborative assessment approach from around 2014 (Jenkins 2019). These reforms were guided by a series of Freshwater Policy Statements prepared at a national level, including explicit standards for water quality, such as levels of nitrogen or pathogens in surface and ground water.

Notwithstanding these efforts, large areas of the plains, their people and communities continue to experience elevated levels of nitrogen and *E. coli* in groundwater, directly attributed to agricultural intensification, alongside historical, high, phosphorous levels and sedimentation, including from pastoral agriculture (Knottenbelt 2023, Snelder et al. 2023). There are resulting challenges for developing environmental limits with agreed thresholds in line with national standards, and associated regulations around land use, with monitoring against bottom lines across the region (Snelder et al. 2023; Jenkins 2018). Of particular concern is how regulators understand and weigh competing outcomes from agricultural uses of water (Prickett et al. 2024), given the different and competing impact pathways and outcomes for social wellbeing that present to them.

## Social Consequences of Cumulative Effects

As Ehrlich (2022) points out, this type of complexity is due to the combination of cumulative effects with past, present and future components and requires integrated thinking. In particular, the Canterbury example requires well integrated social analysis. The region highlights the social consequences of cumulative environmental impacts whereby impact chains result in multiple impact pathways that affect people and communities. The combined impact chains, over time, have potential positive and negative implications for health and wellbeing outcomes. To understand these impact chains and their social impacts we drew on a comparative, retrospective review of six strategic SIAs of water regulation (Mackay and Taylor 2020), of which five were based in the Canterbury Region. In these five SIAs, options for change were explored for their potential social impacts in a collaborative process of impact assessment where scenarios of change were set against the baseline projected into the future, and then scored against social-economic, ecological, and cultural outcomes listed by catchment stakeholders. The social impact assessments drew on ex post analysis of social change from a number of different studies, including academic research, along with primary research using interviews and participation in community engagement processes. Each SIA was conducted alongside physical catchment modeling, in conjunction with ecological, economic, and cultural assessments (Taylor and Mackay 2016). No new research was undertaken during the comparative review, but reference is made to recent publications with findings relevant to the content of the SIAs and the understanding of cumulative effects.

The complexity in the assessments comes from a combination of positive and negative outcomes for human wellbeing, where the outcomes were assessed against criteria established through community involvement. An integrated approach facilitated the work in the technical teams where the SIA practitioners looked to the technical experts in other fields (groundwater, water quality, hydrology, ecology, economics) to help understand potential causes of social impacts from cumulative environmental effects. This work required active dialog from the scoping stage (Ehrlich 2022) to understand, for example, how nitrogen in waterways could affect ecological outcomes and how these, in turn, could affect water users such as outdoor recreationalists or cultural users (Mackay et al. 2024). Exploration of these impact chains was done in the SIAs using webbing and chaining diagrams (after Taylor et al. 2004).

**Fig. 2:**
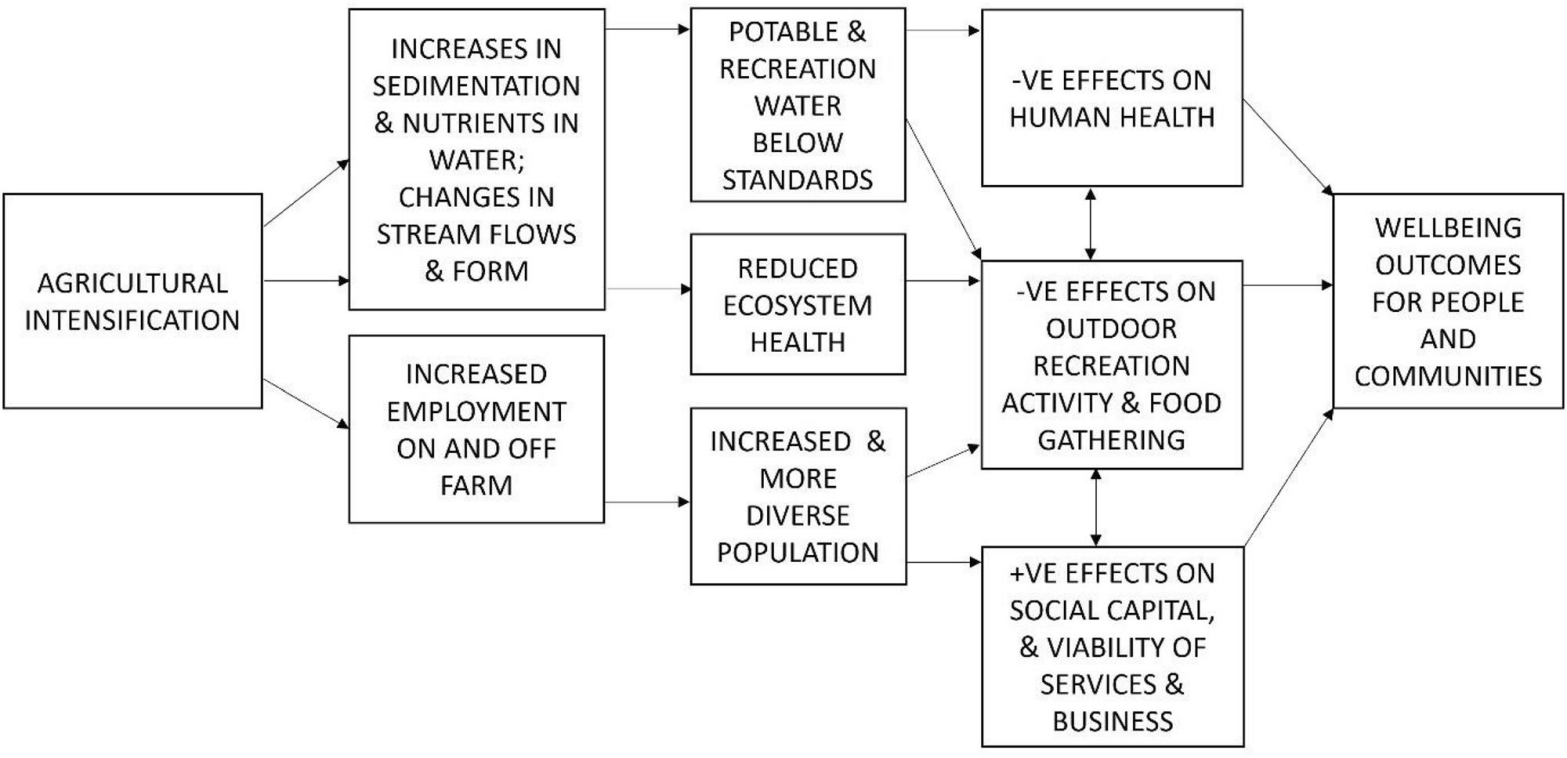
Effects of intensive agriculture on people and communities: summary diagram

Across the five Canterbury SIAs we found complex, multiple, impact pathways from the cumulative effects of agricultural intensification on water quality affecting people and communities and their local economic, public health, recreational, and cultural outcomes. Figure 2 provides a composite, summary description of the links between agricultural intensification and social wellbeing. The pathways in the summary diagram are explored further below it, including webbing effects that cross between chains.

### Health Effects

The cumulative effects of irrigation across multiple farms have resulted in an increase in pathogens and nutrients in groundwater (Knottenbelt 2023), which affects the quality of potable water in household and community bores and water supply takes from rivers, with impacts on human health across the households and community water supplies of the region. The SIAs noted potable water that meets recognized national and international standards is a basic human right and therefore a fundamental part of the wellbeing of people and communities. Direct impacts on human health can result from an increase in nitrates or pathogens in drinking water, and these have included community outbreaks of gastro-intestinal disease in some communities and increased risk of cancers (Green 2014). Most Canterbury communities face additional costs to households and settlements for improved supplies such as deeper wells, chlorination, and other treatments (Prickett et al. 2024).

Other impact chains link to human health. For instance, there are indirect beneficial effects on the physical and mental health of rural households from reduced vulnerability of farmers and farm workers to periods of stressful droughts. There is also a link between the level of farm income, associated living standards, and the mental and physical health of rural people. Furthermore, the size of rural populations affects the quality and level of access to health services (primary and emergency response) and their largely population-based funding (Mackay and Taylor 2020).

The SIAs found further chains of impacts on human health, from the level of wild food collection and recreation activities in waterways. The quality of surface water has a direct effect on the health of humans and their pets during water-based recreation when people or pets have exposure to pathogens (*E. coli* indicators), or cyanobacteria toxins that result from high nutrient values in waterways. Monitoring in recent years has found sites in lower reaches, estuaries and along the coasts have breached standards (Arthur 2020) and these sites are often close to population centers and therefore close to demand for outdoor recreation. Communities have experienced frequent warnings about the risks to human and animal health through water quality readings available online.

Reduced recreational activity can occur due to actual increases in disease experienced due to contact with water, as well as from perceived risks from contact with water and direct warnings about poor quality of water. These all can impact human health negatively as outdoor recreation is part of a healthy lifestyle for New Zealanders, providing opportunities for physical exercise and associated health benefits, rest, enjoyment of nature, enhanced self-worth, and community identity (Angus and Associates, 2017).

### Recreation Effects

Overall, the SIAs identified that the Canterbury region has experienced widespread declines in outdoor recreational activity based on waterways, even allowing for societal changes in patterns of outdoor recreational activity. Increased sedimentation in lowland streams, lakes, and estuaries, along with ecological changes with fewer macrophytes, increased periphyton and phytoplankton, and fewer invertebrates and fish, have reduced ecosystem health. There is a consequent reduction in water-based recreation associated with the social and cultural values of freshwater, when water is visually unappealing and when warnings are in place, including passive and active recreation such as walking, picnicking, swimming, boating, angling, food gathering, and bird watching. Another factor is change in access to waterways from intensive land uses that affect the willingness of landowners to allow access to waterways across farms, with physical barriers such as fences and fewer public physical access points. Finally, there is a flow on effect from outdoor recreation to businesses from commercial recreation activity and tourist operations, and to businesses that support these operations.

### Cultural Effects

Indigenous communities have direct relationships between their mana (prestige), mauri (life forces) and hauora (mental, physical and spiritual wellbeing) and the quality of freshwater (Wehi et al. 2019). The assessments of social impacts, alongside assessments of impacts on cultural values, indicate that waterways are home to taonga (valued components), such as species that are important sources of culturally based food gathering (mahinga kai) including eel species, freshwater crayfish, whitebait and freshwater mussels. In addition, estuaries and lagoons, many now highly degraded, are home to important migratory fish and shellfish used for food and cultural practices. Families (whānau) and subtribes (hapū) recognize the health of mahinga kai as a fundamental indicator of the health of waterways. The quality of water and ecosystem health available for mahinga kai has a direct effect on those who gather foods for consumption or cultural uses, and an effect on the mana and mauri of people associated with the affected water (Tipa & Associates 2013).

### Economic and Social Effects

It is important to recognize that alongside largely negative cumulative effects on social and cultural values, the Canterbury SIAs also identified positive social-economic impacts of agricultural intensification, as shown in Fig. 2. These result from new economic activity on farms and greatly increased farm outputs, and from off-farm activities (transport, farm supplies, processing, and their direct and induced impacts). There were flow-on effects to increased and more regular employment on and off farms, with increases in household incomes and regional GDP. An increase in market opportunities and local entrepreneurship resulted. The SIAs found that these changes typically flow into an increase in total population and a younger age structure as new workers moved in. Many workers are new migrants, such as Filipino farm workers, and they bring an increase in cultural diversity and vibrancy to communities when supported by social impact management practices. Local school rolls grow and there is an increase in demand for worker housing along with a boost in demand for social services in small rural communities previously experiencing population decline and ageing.

A final observation in this example is that the SIAs found many local, community-based initiatives to restore the health of streams and waterways involving farmer catchment groups, irrigation companies, the central government Department of Conservation, regional and local councils, Indigenous groups, recreation and tourism interests, and community groups. Their actions include environmental monitoring, riparian planting and wetland restoration, predator control and wildlife management. There is also an important link between freshwater conservation actions and environmental education, with local schools often involved in this work. These collective actions enhance community resilience and cohesion and build the social license to operate between farming and diverse urban interests.

We emphasize that this example of aggregating cumulative impacts from multiple causal pathways because of intensive agricultural development is retrospective. Recognition of cumulative effects was implicit in the planning effort that the five SIAs were part of but, on reflection, required more explicit analysis as part of a strategic response by relevant authorities under the RMA. The collaborative planning process had many elements of hierarchical SEA as practiced internationally: national-level direction to set limits with agreed thresholds, through Freshwater Policy Statements: assessment and planning by regional government: collaboration with Indigenous groups and inputs from other government agencies such as conservation and public health, local government, and NGOs (Morgan and Taylor 2021). Explicit analysis of cumulative effects in an integrated approach from the start could have helped planners to balance diverse, variable outcomes from new policy and regulations and enhance SEA practice.

## Conclusions

The water management strategic assessment example in this paper shows, at a regional level, the importance of linking cumulative environmental effects to their social consequences, as per Arnold et al. (2024). The example tracks the social impacts of intensive agriculture from effects on water quality and freshwater ecology to effects on drinking water, amenity values, outdoor recreation, and cultural practices. There are also cumulative economic and social impacts for communities. Our reflections on the SIAs undertaken found that the challenge of better water management is endlessly complex as it touches many aspects of social life and creates important social choices, requiring an integrated approach to impact assessment (Mackay et al. 2024).

We found similar projects or developments causing multiple, similar effects, locally or regionally, require better strategic thinking from planning and consent authorities. This problem appears particularly the case for NZ, where there is considerable potential to improve processes for assessments of cumulative effects, through improvements in the institutional framework as well as in the theory and practice of SEA (Morgan and Taylor 2021).

The example of land use change shows it is possible to improve practice in the assessment of cumulative effects by tracking systematically how environmental impacts have social consequences. A simplistic approach to cumulative effects is to consider them when environmental monitoring shows the buildup of an environmental impact has reached a limit or threshold, such as levels of nitrogen or pathogens in surface or ground water. Another approach is to consider a cumulative effect when it has obvious social or cultural consequences, such as the loss of potable water or the ability to recreate or collect food from waterways. But effects such as decreased water quality often have an historical lag, for instance the slow movement of nutrients through groundwater in a catchment, with delayed effects on freshwater quality and consequential effects on people, cultural values and human health over time. The point that a threshold is breached (as defined by an agreed indicator) such as potable water that meets drinking water standards or a notification not to collect food, or to swim at a popular site, represents a level of complexity not always accounted for in standard impact assessment practice. In a practical sense this requires specific steps to overcome practice silos (Ehrlich 2022), especially for SIA. In a theoretical sense, system complexity requires integrating SIA better into collaborative processes, with a greater focus on social-cultural outcomes (Morgan 2022; Mackay et al. 2024).

Looking ahead, the unclear mandate for SEA in NZ requires specificity in legislative revisions, consistent with SEA and CEA theory and practice. The impact assessment community needs to ensure that the assessment of cumulative effects at project and strategic levels is part of future legislative changes. There is considerable potential here to build on the base of case law, impact assessment research, practice experiences, and practical guidelines, in NZ and internationally.

## Data Availability

No datasets were generated or analysed during the current study.

## References

[CR1] Angus and Associates (2017) The value of sport and active recreation to New Zealanders. Report commissioned by Sport NZ. https://sportnz.org.nz/resources/the-value-of-sport/.

[CR2] Arnold L, Hanna K, Fell C, LaPlante, JP, Nishima-Miller J, Wade J (2023) Incorporating social impacts into cumulative effects assessment: lessons and best practices. Tŝilhqot’ in National Government, Nen (Water, Lands, and Resources) Department and the University of British Columbia’s Centre for Environmental Assessment Research.

[CR3] Arnold LM, Hanna K, Fell C, Laplante JP (2024) Addressing cumulative effects through an Indigenous-led assessment process. Environ Manag. Published online. 10.1007/s00267-024-02084-z. 10.1007/s00267-024-02084-z 39562362

[CR4] Arthur J (2020) Canterbury water quality monitoring for primary contact recreation Annual Summary Report 2019/20. Report No. R20/56, Environment Canterbury, Christchurch.

[CR5] Baines J, Taylor CN (2021) Green is good, but is more green always better? Wind farm development and cumulative social impact assessment. In Blakely, JAE, Franks DM, (eds) Handbook of Cumulative Impact Assessment. Edward Elgar Publishing, Cheltenham, pp 213-229. 10.4337/9781783474028.00025.

[CR6] Blakely JAE, Franks DM (2021) Handbook of Cumulative Impact Assessment. Edward Elgar Publishing, Cheltenham. 10.4337/9781783474028.

[CR7] Borthwick J (2017) In the matter of Yaldhurst Quarries Joint Action Group, Christchurch City Council and Harewood Gravels. Decision 2017/165 NZ EnvCourt. https://www.disputestribunal.govt.nz/assets/2017-NZEnvC-165-Yaldhurst-Quarries-Joint-Action-Group-v-Christchurch-City-Council.pdf.

[CR8] Bragagnolo C, Geneletti D, Fischer TB (2012) Cumulative effects in SEA of spatial plans–evidence from Italy and England. Impact Assess Proj Apprais 30(2):100–110. 10.1080/14615517.2012.677522.

[CR9] Burdge R (2004) Identifying social impact assessment variables. In Burdge R (ed) The Concepts, Process and Methods of Social Impact Assessment. Social Ecology Press, Wisconsin, pp 41-52.

[CR10] Canter L, Ross B (2010) State of practice of cumulative effects assessment and management: the good, the bad and the ugly. Impact Assess Proj Apprais 28(4):261–268. 10.3152/146155110X12838715793200.

[CR11] Davies K, Fisher K, Foley M, Greenaway A, Hewitt J, Le Heron R, Mikaere H, Ratana K, Spiers R, Lundquist C (2018) Navigating collaborative networks and cumulative effects for sustainable seas. Environ Sci Policy 83(May):22–32.

[CR12] Ehrlich A (2022) Collective impacts: using systems thinking in project-level assessment. Impact Assess Proj Apprais 40(2):129–145. 10.1080/14615517.2021.1996901.

[CR13] Grace B, Pope J (2021) A systems approach to cumulative social impact assessment. In: Blakley JAE, Franks D.M (eds) Handbook of Cumulative Impact Assessment, Edward Elgar Publishing, Cheltenham, pp 174–189. 10.4337/9781783474028.00022.

[CR14] Green J (2014) Public health implications of land use change and agricultural Intensification with respect to the Canterbury Plains. A Literature Review, Canterbury, District Health Board, Christchurch. https://www.cph.co.nz/wp-content/uploads/landusechangehealthreview.pdf.

[CR15] IAIA (2017) Cumulative Effects Assessment. Fastips 16. https://www.iaia.org/uploads/pdf/Fastips_16%20Cumulative%20Effects%20Assessment_1.pdf.

[CR16] Jenkins B (2018) Challenges in cumulative impact assessment: case studies from Canterbury, New Zealand. https://www.witpress.com/elibrary/wit-transactions-on-ecology-and-the-environment/215/36698.

[CR17] Jenkins B (2019) Changing water management practice in Canterbury to address sustainability limits. Policy Q 15(9):29–36. 10.26686/pq.v15i3.5685.

[CR18] Jones CF (2015) Cumulative effects assessment: theoretical underpinnings and big problems. Environ Rev 24:187–204. 10.1139/er-2015-0073.

[CR19] Knottenbelt M (2023) Annual groundwater quality survey 2023. Environment Canterbury Regional Council, Christchurch. https://www.google.com/search?client=firefox-b-d&q=Annual+groundwater+quality+survey+2023.+Environment+Canterbury+Regional+Council%2C+Christchurch.

[CR20] Loxton EA, Schirmer J, Kanowski P (2013) Exploring the social dimensions and complexity of cumulative impacts: a case study of forest policy changes in Western Australia. Impact Assess Proj Apprais 31(1):52–63. 10.1080/14615517.2012.755353.

[CR21] Mackay M, Taylor CN (2020) Understanding the social impacts of freshwater reform: A review of six limit setting social impact assessments. AgResearch Report RE450/2020/005 for New Zealand Ministry for the Environment, AgResearch Lincoln Research Centre, Christchurch, New Zealand. https://environment.govt.nz/assets/Publications/Files/understanding-social-impacts-freshwater-reform.pdf

[CR22] Mackay M, Taylor CN, Saunders J, Rutherford P, Saunders C (2024) Integrated impact assessment for land and water management. Environ Impact Assess Rev 105: 107397. 10.1016/j.eiar.2023.107397.

[CR23] MacLeod CJ, Moller H (2006) Intensification and diversification of New Zealand agriculture since 1960: An evaluation of current indicators of land use change. Agric Ecosyst Environ 115(1-4):201–218. 10.1016/j.agee.2006.01.003.

[CR24] Malakar Y, Peeters L, Walton A, O’Sullivan D (2023) A causal network approach using a community well-being framework for an initial impact assessment of large-scale energy infrastructure projects. Environ Impact Assess Rev 102: 107188. 10.1016/j.eiar.2023.107188.

[CR26] Ministry for the Environment and Statistics NZ (2022) New Zealand’s Environmental Reporting Series: Environment Aotearoa 2022. Ministry for the Environment and Stats NZ, Wellington. https://environment.govt.nz/assets/publications/Environmental-Reporting/environment-aotearoa-2022.pdf

[CR27] Milne P (2008) When is enough, enough? Dealing with cumulative effects under the Resource Management Act. https://www.qualityplanning.org.nz/sites/default/files/When_is_Enough_Enough_Dealing_with_Cumulative_Effects_Under_the_RMA.pdf

[CR28] Morgan RK (2012) Environmental impact assessment: the state of the art. Impact Assess Proj Apprais 30(1):5–14. 10.1080/14615517.2012.661557.

[CR29] Morgan RK (2022) Integrated impact assessment: coming out of the shadows? In: Fonseca A (ed) Handbook of Environmental Impact Assessment. Edward Elgar Publishing, Cheltenham, pp 66-83. 10.4337/9781800379633.00009

[CR30] Morgan RK, Taylor CN (2021) Strategic environmental assessment in New Zealand. In: Fischer TB, González A (eds) Handbook of Strategic Environmental Assessment. Edward Elgar Publishing, Cheltenham pp. 332-348. 10.4337/9781789909937.00035

[CR31] Noble B (2008) Strategic approaches to regional cumulative effects assessment: a case study of the Great Sand Hills, Canada. Impact Assess Proj Apprais 26(2):78–90. 10.3152/146155108X316405.

[CR32] Olagunju A, Appiah DO, Maria P, Cavalcanti PS, Durning B, Tejeda-González JC, McLean J, Morgan RK, Nelson R (2021) Cumulative effects assessment requirements in selected developed and developing countries. In: Blakley, JAE, Franks DM (eds) Handbook of Cumulative Environmental Assessment. Edward Elgar Publishing, Cheltenham, pp. 21-42. 10.4337/9781783474028.00012

[CR33] Pangborn M, Woodford K (2011) Canterbury dairying - a study in land use change and increasing production. Proceedings of the 18th International Farm Management Congress, Methven, Canterbury, New Zealand

[CR34] Peeters LJM, Holland KL, Huddlestone-Holmes C, Boulton AJ (2022) A spatial causal network approach for multi-stressor risk analysis and mapping for environmental impact assessments. Sci Total Environ 802:149845. 10.1016/j.scitotenv.2021.14984510.3152/146155108X316405. 34455278 10.1016/j.scitotenv.2021.149845

[CR35] Prickett M, Canning A, Chambers T, Baker M, Hales S (2024) Regulator failure on nitrate in drinking water dumps escalating costs on those downstream. Public Health Expert Briefing. https://www.phcc.org.nz/briefing/regulator-failure-nitrate-drinking-water-dumps-escalating-costs-those-downstream

[CR36] Slootweg R, Vanclay F, van Schooten M (2001) Function evaluation as a framework for the integration of social and environmental impact assessment. Impact Assess Proj Apprais 19(1):19–28. 10.3152/147154601781767186.

[CR37] Snelder T, Smith H, Plew D, Fraser C (2023) Nitrogen, phosphorus, sediment and Escherichia coli in New Zealand’s aquatic receiving environments: Comparison of current state to national bottom lines. LWP Client Report 2023-06, November 2023. https://ourlandandwater.nz/outputs/comparison-of-current-state-to-nbls/

[CR38] Taylor, CN, Goodrich, CG, Bryan, CH, 2004. Social Assessment: Theory, Process and Techniques. Social Ecology Press, Middleton, Wisconsin.

[CR39] StatsNZ (2024a) Irrigated land: Data to 2022 (updated 2024). https://www.stats.govt.nz/indicators/irrigated-land-data-to-2022/

[CR40] StatsNZ (2024b) Livestock numbers: Data to 2023 (updated 2024). https://www.stats.govt.nz/indicators/livestock-numbers-data-to-2023/

[CR41] Taylor CN (2017) Evidence on social impacts to Borthwick J in the matter of Yaldhurst Quarries Joint Action Group, Christchurch City Council and Harewood Gravels. Decision 2017/165 NZ EnvCourt

[CR42] Taylor CN, Mackay M (2016) Practice issues for integrating strategic social assessment into the setting of environmental limits: insights from Canterbury, New Zealand. Impact Assess Proj Apprais 34(2):110–116. 10.1080/14615517.2016.1147261.

[CR43] Taylor CN, Mackay M (2022) Social Impact Assessment: Guidelines for Thriving Regions and Communities. Wellington: Building Better Homes, Towns and Cities National Science Challenge, Wellington. New Zealand, https://www.buildingbetter.nz/toolkit/social-impact-assessment-guidelines-for-thriving-regions-and-communities/

[CR44] Tipa & Associates (2013) Cultural values, flow & water management issues for the Waikirikiri / Selwyn-Te Waihora catchments. Report to Environment Canterbury, Christchruch

[CR45] Wehi P, Beggs JR, McAllister TA (2019) Ka mua, ka muri: the inclusion of mātauranga Māori in New Zealand ecology. N Zeal J Ecol 43(3):3379. 10.20417/nzjecol.43.40.

